# Studies on the Cytotoxic Activities of *Punica granatum* L. var. *spinosa* (Apple Punice) Extract on Prostate Cell Line by Induction of Apoptosis

**DOI:** 10.5402/2012/547942

**Published:** 2012-12-17

**Authors:** Koushan Sineh Sepehr, Behzad Baradaran, Masoumeh Mazandarani, Vahid Khori, Fatemeh Zare Shahneh

**Affiliations:** ^1^Immunology Research Center (IRC), University of Medical Sciences Tabriz, Iran; ^2^Department of Botany, Gorgan Branch, Islamic Azad University, Gorgan, Iran; ^3^Golestan Physiology Pharmacology Center, Golestan University of Medical Sciences, Gorgan, Iran

## Abstract

The *Punica granatum* L. var. *granatum* (pomegranate) has been demonstrated to exert antitumor effects on various types of cancer cells. The present study aimed to evaluate the medicinal herbs *Punica granatum* L. var. *spinosa* (apple punice) that are native to Iran. This study was determined to test the possible cytotoxic activity and induction of apoptosis on human prostate cell lines. The effect of ethanol extracts of the herbs on the inhibition of cell proliferation was assessed by MTT colorimetric assay. PC3 cell lines treated with the extracts were analyzed for the induction of apoptosis by cell death detection (ELISA) and TUNEL assay. Dye exclusion analysis was performed for viability rate. Our results demonstrated that the *Punica granatum* L. var. *spinosa* extract dose dependently suppressed the proliferation of PC3 cells (IC_50_= 250.21 **μ**g/mL) when compared with a chemotherapeutic anticancer drug (Toxol) (Vesper Pharmaceuticals) with increased nucleosome production from apoptotic cells. The *Punica granatum* L. var. *spinosa* extract attenuated the human prostate cell proliferation *in vitro* possibly by inducing apoptosis. The *Punica granatum* L. var. *spinosa* is likely to be valuable for the treatment of some forms of human prostate cell line.

## 1. Introduction

Apoptosis (programmed cell death) is a physiological mechanism of cell death. During apoptosis, there is a rapid reduction in the cellular volume followed by chromatin condensation, associated with characteristic internucleosomal DNA cleavage. This results in the production of nucleosomes of DNA fragments complexes with core histones, which are distinct multiples of an 180–200 bp subunit [1]. Cancer is one of the major causes of mortality throughout the world. World Health Organization statistics have estimated that cancer will cause 83.2 million deaths between 2005 and 2015 if the recommended measures are not respected. In 2007, cancer was the cause of 7.9 million deaths, which is 13% of world mortality. Among males in the third world countries, prostate cancer is the second-leading cause of cancer-related death [2]. Cancer is a disease that is characterized by too little apoptosis. Under normal circumstances damaged cells will undergo apoptosis, but in the case of cancer cells mutations may have occurred that prevent cells from undergoing apoptosis. Understanding apoptosis regulation is a main concern in the development of chemotherapeutic anticancer drugs on malignant cells [3, 4]. Traditionally, many extracts from roots, stems, and fruits have been used for maintaining health, enhancing overall immune status, and prevention and treatment of chronic diseases, and the modulation and treatment of different diseases [5]. 

*Punica granatum *L. var.* spi*nosa is known as apple punice from Punicaceae family commonly widespread in the latitudes of 475 m above sea level in the north of Iran. Recent studies have demonstrated that *Punica granatum *L. var.* granatum *extracts possess a plethora of biological activities including antibacterial, antiviral, antifungal, cytotoxic and immuno-potentiating activities. The *Punica granatum *L. var.* granatum* tree (pomegranate), especially its fruit, possesses a vast ethno medical history and represents a phytochemical reservoir of heuristic medicinal value. The tree/fruit can be divided into several anatomical compartments: (1) seed, (2) juice, (3) peel, (4) leaf, (5) flower, (6) bark, and (7) roots, each of which has interesting pharmacologic activity. Juice and peels, for example, possess potent antioxidant properties, while juice, peel, and oil are all weakly estrogenic and heuristically of interest for the treatment of menopausal symptoms. The use of juice, peel, and oil has also been shown to possess anticancer activities, including interference with tumor cell proliferation, cell cycle, invasion, and angiogenesis [6]. The toxicity of *Punica granatum *L. var.* spinosa* has not been intensively studied. Accordingly, we have conducted our research on toxicity extracts of *Punica granatum *L. var.* spinosa* seeds and peels [7, 8]. The objective of this study was to examine the *in vitro* cytotoxic activities of a wildly ethanolic standardized *Punica granatum *L. var. *spinosa *(PGS) extract using a MTT cytotoxicity assay. The study also tested whether the mechanism of action involves induction of apoptosis. Cell death ELISA and TUNEL was employed to quantify the nucleosome production resulting from nuclear DNA fragmentation during apoptosis.

## 2. Materials and Methods

### 2.1. Preparation of Plant Extract

*Punica granatum *L. var.* spinosa *(PGS) plants known as apple punice from Punicaceae family were collected from the southeast of Golestan province, Iran (Ramian). Dr. Mazandarani from the Medicinal Plant Research Center of Islamic Azad University of Gorgan, Iran, identified the plant. A voucher specimen was deposited in the herbarium of the above mentioned (no. 315HRCMP). The seeds and peels parts of the plant were separated, shade dried, and grinded into powder with mortar and pestle. The prepared powder was kept in tight containers protected completely from light. Extraction of ethanolic extract was carried out by macerating 100 g of powdered dry plant in 500 mL of 70% ethanol for 48 h at room temperature. Then, the macerated plant material was extracted with 70% ethanol solvent by percolator apparatus (2-liter volume) at room temperature. The plant extract was removed from percolator, filtered through Whatman filter paper (NO. 4), and dried under reduced pressure at 37°C with rotator evaporator. The ethanol extract was filtered and concentrated using a rotary evaporator and then evaporated to dryness. Briefly, the concentrated plant extracts were dissolved in dimethyl sulphoxide (DMSO) (SIGMA, USA) to get a stock solution of 10 mg/mL. The substock solution of 0.2 mg/mL was prepared by diluting 20 *μ*L of the stock solution into 980 *μ*L serum-free culture medium, RPMI 1640 (the percentage of DMSO in the experiment should not exceed 0.5). 

### 2.2. Cell Cultures

The human prostate cancer cell line (PC3) and normal fibrosarcoma cell line (L929) were obtained from National Cell Bank of Iran (NCBI, Pasteur Institute of Iran). The cells were grown and maintained in a humidified incubator at 37°C and in 5% CO_2_ atmosphere. RPMI-1640 medium supplemented with 10% fetal bovine serum (FBS, Invitrogen Gibco), 100 units/mL penicillin, and 100 *μ*g/mL streptomycin (Invitrogen Gibco) was used for cell cultures of PC3. Ten thousand cells from log phase cultures were seeded in 100 *μ*L of RPMI-164 medium supplemented with 10% fetal bovine serum per well of 96-well flat-bottom culture plates (Nunc, Denmark). Proliferative response and cell death of the PGS extract-treated cells were determined using MTT assay and cell death ELISA, respectively [9].

### 2.3. MTT Colorimetric Assay

A colorimetric assay using 3-(4, 5-dimethylthiazoyl)-2, 5-diphenyltetrazolium bromide (MTT) was performed. Briefly, cells were added onto flat-bottomed microculture plates in the presence or absence of various concentrations of the extracts (in triplicate) and incubated at 37°C in a 5% humidified CO_2_ incubator for 24 and 48 h. Then, 10 mL of MTT (5 mg/mL, Sigma) was added to each well and incubation was continued for a further 4 h at 37°C. In each well, 100 *μ*L/well of solubilization solution, containing DMSO and Sorenson buffer, were added. After complete solubilization of the dye, plates were read at 570 nm on an ELISA reader. The mean optical density (OD) ± SD for each group of replicates was calculated. The whole procedure was repeated for three times. The inhibitory rate of cell growth was calculated using the formula: % Growth inhibition = (1− OD extract treated)/OD negative control × 100 [9].

### 2.4. Cell Death Detection

cell death detection ELISA^PLUS^ (Roche Applied Science, Switzerland) was used to quantify histone-complexed DNA fragments (nucleosomes) in cytoplasm of the apoptotic cells after induction of apoptosis, as described elsewhere [10]. Briefly, after incubation with the PGS extract (at concentrations determined by MTT assay) for 24 h, the PC3 cells were pelleted and lysed. The remaining steps were carried out according to the instructions supplied by the manufacturer. The resulting color development, which was proportional to the amount of nucleosomes captured in the antibody sandwich, was measured at 405 nm (with reference wavelength at 490 nm) using a Benchmark microtiter plate reader (Bio-Rad). Results were expressed as the apoptotic and necrosis percentage, calculated from the ratio of absorbance of treated (apoptotic) sample to that of the untreated (control) sample [10].

### 2.5. Dye Exclusion Assay

Viability induced of the PGS extract treatment was measured using trypan blue exclusion assay. Briefly, 1 × 10^4^ cells were seeded into 96-well plates and treated with or without (as control) PGS extract at specified doses for 24 h. After the incubation period, the cultures were harvested and washed twice with PBS. The cell pellet was then resuspended with 0.5 mL PBS. Then, 20 *μ*L of cell was mixed with equal volume of 0.4% trypan blue (Sigma, USA Merck) and was count with Neubauer haemocytometer (Weber, England) by clear field microscopy (Nikon, japan). Only viable cells were counted. Each extract and control were assayed two times in triplicate [10].

### 2.6. Apoptosis Assay

To assess cell death by apoptosis, an In Situ Cell Death Detection Kit, POD (Roche, Germany) for DNA chromatin morphologic features was used for quantification. The procedures followed the manufacture's guidelines. Briefly, cells were cultured on glass slides and analyzed 24 hours after treatment. Cells grown on coverslips were washed twice with PBS, air dried, and fixed for 60 min in freshly prepared 4% paraformaldehyde/PBS (pH: 7.4) (Sigma-Germany), pH 7.4, at room temperature. Then the cells were washed again twice with PBS (pH: 7.4) and incubated with 3% H_2_O_2_/methanol (Merck-Germany) for 10 min. Following washing with PBS, cells were permeabilized in 0.2% Triton X-100/PBS (pH: 7.4) (Sigma-Germany) for 2 min at 4°C. Samples were incubated in 50 *μ*L of TUNEL reaction mixture for 2 h at 37°C in a humidified chamber and in the dark, covered with parafilm. Omission of TdT provided the negative control for the assay, and preincubation of cells with 10 *μ*g/mL DNase I in 50 mM Tris-HCl, pH 7.4, 1 mM MgCl_2_, and 1 mg/mL BSA for 10 min at room temperature to induce DNA strand breaks artificially, served as positive control. Cells were washed with PBS (pH: 7.4) and incubated for 30 min in a humidified chamber, at 37°C with 50 *μ*L converter-POD (Anti-fluorescein antibody, Fab fragment from sheep, conjugated with horse-radish peroxidase). After rinsing in PBS, the samples were incubated for 10 min with 100 *μ*L DAB (Sigma-Germany) substrate in the dark. At the end, the samples were mounted and analyzed under light microscope, where the apoptotic cells could be seen as condensed shrinked dark brown cells [10].

### 2.7. Statistical Analysis

The data are expressed as mean ± standard deviation (SD) for at least three independent determinations in triplicate for each experimental point. The data were analyzed using IBM SPSS Statistics 20 software. For all the measurements, Tow-way ANOVA followed by Duncan's New Multiple Range Test (*P* ≤ 0.05) was used to assess the statistical significance of difference between control and PGS treated.

## 3. Results

### 3.1. Effects of *Punica granatum* L. var. *spinosa* Extract on Proliferation of Prostate Cancer Cell Line (PC3)

**Figure 1:**
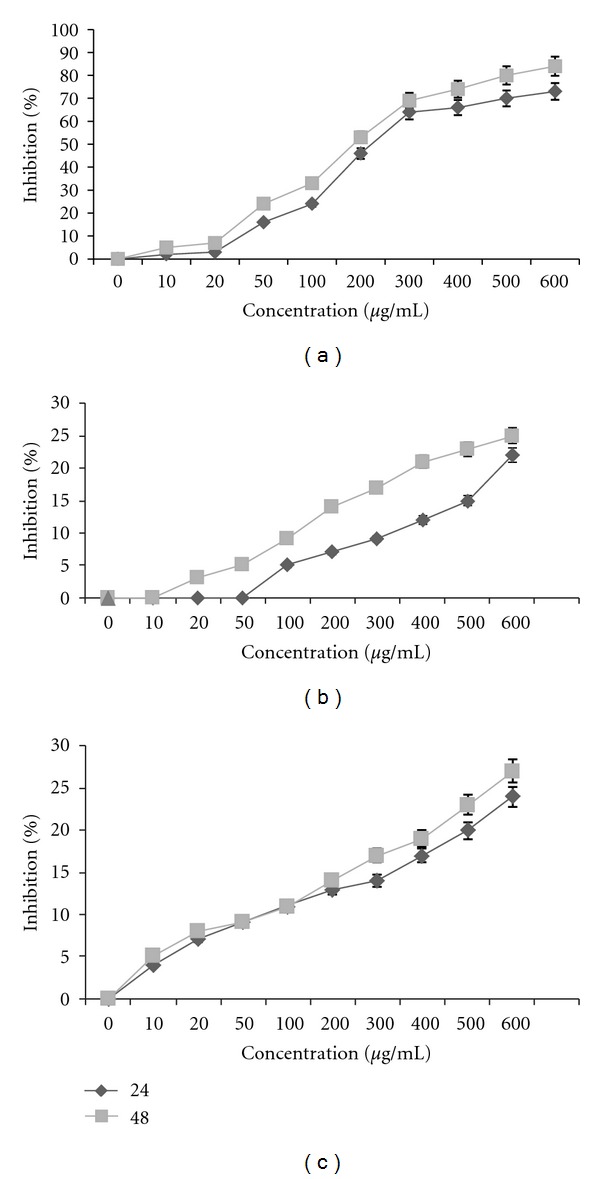
Effects of *Punica granatum spinosa* peel extract with increasing concentrations (10–600 *μ*g/mL with) on proliferation of PC3 cells (a), seed extract on PC3 cells (b), and peel extract on L929 cells (c) for 24 h and 48 h the proliferative response was assessed by MTT assay.

Peels of PGS extract at 10 to 600 *μ*g/mL exhibited significant dose-dependent inhibitory effects on the proliferation of PC3. Growth inhibition of peel extract in 24 and 48 h was 61.2 ± 2.3% and 67.±1.75%, respectively (Figure 1(a)), with more than 75% suppression. The concentrations producing 50% growth inhibition (IC_50_) of the PGS extract on PC3 were effectively suppressed with the IC_50_ value (250.21 *μ*g/mL) after incubation with the peel extract. However seed extract induced no significant suppression on the proliferation of PC3 cells (Figure 1(b)) and the peel extract induced no significant suppression on the proliferation of normal L929 cells (Figure 1(c)). PC3 cells were compared to elucidate the cytotoxicity of both peels of PGS extract and Toxol (chemotherapeutic agent, control positive) with more than 75% in 600 *μ*g/mL and 90% in 20 *μ*g/mL growth suppression in 24 h (Table 1).

**Figure 2:**
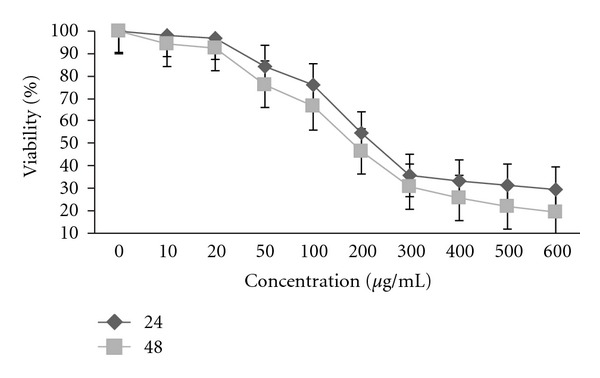
Results are expressed as the mean percentage of viable cells with 3 wells each. Percentage of viable cells was calculated from the ratio of viable cells to total number of cells using trypan blue exclusion test.

In 24 and 48 h Dye exclusion assay evaluated viability of PC3 cells exposed to peel extract. As the result in Figure 2, the viabilities of cells exposed to peel PGS extract at concentrations of 10 and 600 *μ*g/mL were 96.3 ± 7.8% and 24.1 ± 2.5%, respectively. 

### 3.2. Effects of *Punica granatum* L. var. *spinosa* Extract on Cell Death of Prostate Cancer Cell Line (PC3)

As determined by MTT assay, peel extract at 50, 100, 200, and 300 *μ*g/mL was chosen for PC3 cell line in cell death detection ELISA. The proportion of dead PC3 cells increased sharply (from 32 ± 8.5%, to 55 ± 1.9%) upon 24 h incubation with the peel extract at 50–300 *μ*g/mL at 24 h. These results suggested that the apoptotic response of PC3 cell lines should be evaluated at different concentration points. 

### 3.3. *Punica granatum* L. var. *spinosa* Extract Induced Apoptosis in Prostate Cancer Cell Line (PC3)

To test whether or not peel extract that induced the decrease of cell viability and cytotoxicity contributes to apoptotic death in PC3 cell lines *in vitro*. Cells were incubated with 250 *μ*g/mL of PGS for 24 h and then determined using TUNEL assay. It was found that the PC3 cells treated with peel extract (250 *μ*g/mL) for 24 h exhibited apoptotic body formation (Figure 4). PC3 cells treated with peel extract displayed typical morphological features of apoptotic cells, with condensed and fragmented nuclei (Figure 4(a)). However, homogenous nuclear chromatin was evident in control cells (Figure 4(b)). The induction of apoptosis by peel extract was confirmed by in situ TUNEL assay. TUNEL assay based on labeling of DNA strand breaks generated during apoptosis revealed that peel extract induces apoptosis in PC3 cells.

## 4. Discussion

Apoptosis (programmed cell death) is a physiological mechanism of cell death. Cancer is one of the major causes of mortality throughout the world. Cancer is a disease that is characterized by too little apoptosis. Understanding apoptosis regulation is a main concern in the development of chemotherapeutic anticancer drugs on malignant cells [2]. The present study has demonstrated that ethanolic peel extract of PGS in natural form could significantly suppress the proliferation of PC3 cells *in vitro* using the MTT assay. Such antiproliferative activity of peel extract of PGS was characterized by the dose-dependent manner (Figure 1(a)). However seed extract induced no significant suppression on the proliferation of PC3 cells (Figure 1(b)) and the peel extract induced no significant suppression on the proliferation of normal L929 cells (Figure 1(c)). Toxol at an optimal *in vitro* concentration was found to selectively induce at least 90% growth suppression on PC3 cells but peel extract has more than 75.50% in 600 *μ*g/mL in comparison to 90% inhibition activity Toxol in 20 *μ*g/mL growth suppression in 24 h (Table 1). Viability percentage was evaluated by dye exclusion assay at 24 h with peel extract at concentrations of 10 to 600 *μ*g/mL and viable cell decreases from 96.3 ± 7.8% to 24.1 ± 2.5% by increasing dose and time of treatment (Figure 2). 

**Figure 3:**
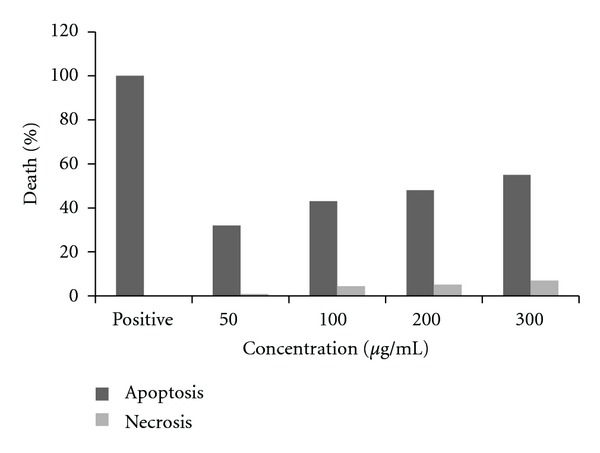
Effects of *Punica granatum spinosa* extract on cell viable of PC3 cells. Cells were incubated with the *Punica granatum spinosa* extract in culture medium at concentrations derived from IC_50_, 50, 100, 200, and 300 *μ*g/mL for 24 h. The induced apoptosis (internucleosomal DNA fragmentation) was then assessed by cell death detection ELISA.

**Figure 4:**
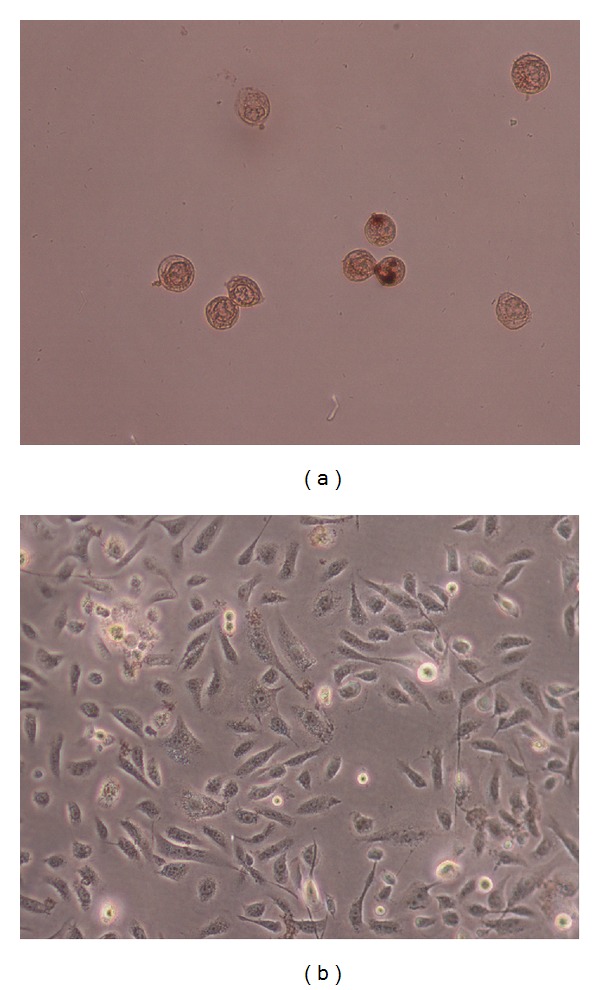
Nuclei morphological changes during *Punica granatum spinosa* induced apoptosis in PC3 cells detected by TUNEL assay. Tumor cells treated with extract (250 *μ*g/mL) were assayed by TUNEL and observed under light microscopy. For PC3 cells group, (a) treated with extract (250 *μ*g) for 24 h and (b) show negative control (without treatment).

In order to determine whether the antiproliferative activity of peel extract is manifested by induction of apoptosis, cell death detection ELISA was employed to quantify the nucleosome production during nuclear DNA denaturation of apoptotic cells (Figure 3), suggesting dose-and time-dependent induction of apoptosis. These results suggested that the extract exerts its cytotoxic effect on prostate cells possibly via an apoptosis-dependent pathway. Apoptosis is an intracellular suicide program possessing morphologic change and biochemical response. It is known that DNA strand breaks occur during the process of apoptosis, and the nicks in DNA molecules can be detected qualitatively through TUNEL assay. In present study, typical apoptotic characteristic TUNEL staining was observed in treated cells (Figure 4).

The effects of pomegranate on prostate cancer have been investigated in the cell culture system, previously. Various preparations of pomegranate were tested on human PC cell growth *in vitro*. Each preparation suppressed prostate cancer cell growth and invasive potential PCa LNCaP, PC-3, and DU 145 cells, whereas normal prostate epithelial cells were significantly less affected [11]. Antiproliferative properties of pomegranate fruit extract (PFE) against human PCa cells were demonstrated by the Vidal et al. in the cell culture system and in a xenograft mouse model. Human PCa PC-3 cells treated with PFE (10–100 *μ*g/mL) for 48 h resulted in a dose-dependent inhibition of cell growth and induction of apoptosis [8].

Taking together, the present study is the first to show toxicity of PGS in malignant cell lines in which apoptosis or programmed cell death play an important role. It could provide further knowledge to mechanisms involved in this toxicity. PGS could be also considered as a promising chemotherapeutic agent in cancer treatment.

## 5. Conclusion

This study provides the evidence that *in vitro* cytotoxic activity of an ethanol standardized extract from wild *Punica granatum *L. var.* spinosa* (PGS) from southeast of Golestan province, Iran (Ramian), was found to dose dependently inhibit the proliferation of prostate cells possibly via an apoptosis-dependent pathway.

## Figures and Tables

**Table 1a:** (a)

Concentration (*μ*g/mL)	Growth inhibition %
600	75.50 ± 2.6
500	72.21 ± 8.1
400	70.67 ±7.8
300	66.83 ± 3.9
200	49.67 ± 6.5

**Table 1b:** (b)

Concentration (*μ*g/mL)	Growth inhibition %
20	93.78 ± 5.7
15	82.86 ± 2
10	71.69 ± 2.8
5	63.88 ± 3.2
3.5	51.79 ± 1.5
